# Associations between cerebral small vessel disease and reduced forced vital capacity and expiratory volume in a general healthy Swedish elder population study–Good Aging in Skåne

**DOI:** 10.1177/13872877251333793

**Published:** 2025-04-23

**Authors:** Sölve Elmståhl, Katarina Ellström, Tomas Månsson, Rani Basna, Arkadiusz Siennicki-Lantz, Kasim Abul-Kasim

**Affiliations:** 1 Department of Clinical Sciences, Division of Geriatric Medicine, Lund University, Malmö, Sweden; 2 Department of Clinical Sciences, Division of Diagnostic Radiology, Lund University, Malmö, Sweden

**Keywords:** Alzheimer's disease, cerebral small vessel disease, epidemiology, lung function, spirometry

## Abstract

**Background:**

Cerebral small vessel disease (CSVD) is one of the most important causes of cognitive decline. Only a few previous studies have evaluated lung function measures in relation to brain neuropathological changes, and even less studies on specific lesions and areas that could shed light on mechanisms of CSVD.

**Objective:**

The aim was to study the association between lung function and CSVD in the general elder population.

**Methods:**

379 participants, aged 72–87 years from the general population study ‘Good Aging in Skåne study (GÅS)’were investigated with a 3 T MRI brain examination and spirometry. Z-scores of FEV_1_ and FVC were calculated using the GLI 2012 equations. Age-adjusted associations between white matter hyperintensities (WMH), medial temporal lobe atrophy (MTA), lacunar infarction, cerebral atrophies and cerebral microbleeds and lung function were calculated and stratified for sex.

**Results:**

Decreased FEV_1_ and FVC z-scores below ≤ −1.0 were both associated with increased risk of WMI and global cortical atrophy. Decreased FVC z-scores were also associated with MTA and lacunar infarction in women and precuneus atrophy in men. The associations for WMH, MTA and lacunar infarctions and higher STRIVE score were noted among women, but not among men. FEV_1_ z scores were not related to diabetes, coronary artery disease or stroke.

**Conclusions:**

Lower lung function was associated to MRI markers of CSVD in this general healthy population, particularly with WMH, especially for women. Although possible shared risk factors exist between lung and heart disease, lung function should be recognized in future studies on CSVD.

## Introduction

The relationship between lung and heart disease, including shared risk factors, has been recognized in several longitudinal studies,^1–3^ although the mechanisms are unclear. Lung function is also a risk factor for cardiovascular disease and mortality in asymptomatic subjects without respiratory disease.^4^

Few studies have evaluated lung function measures in relation to brain neuropathological changes, and the studies have mostly been related to total brain volume, grey and white matter volumes. The associations to specific lesions and areas are even less investigated.^5^ An association between lung and cerebrovascular disease and risk for cerebral small vessel disease (CSVD), especially brain volume and white matter hyperintensities (WMH) volume on MRI, has been suggested from the Cross-Cohort Collaboration Consortium studying brain, gray matter, hippocampal and WMH volume.^6^ Similar findings for WMH and lung function was noted in the ARIC Neurocognitive study.^7^

Recent studies have reported stronger associations for WMH among women compared to men.^8^ A pooled analysis of 15 population-based cohort studies noted similar differences between sexes, with higher WMH burden in older females.^9^

The aim of this study was to determine which MRI related markers of CSVD could be associated with spirometric lung function assessment and covariates in the general older population, and secondly if any gender differences exist.

## Methods

Data was retrieved from the general population cohort study “Good Aging in Skåne” (GÅS), part of the “Swedish National study on Aging and Care” (SNAC),^10–12^ analyzing MRI changes of the brain related to age. A cross-sectional design was used. Subjects were invited by letter after a random selection from the National Population Register to an out-patient research center. Results on prevalence of CSVD at the baseline examination has been presented by us previously.^10^ Of the 407 eligible persons with MRI brain from the study baseline invited in 2015 to 2017, 379 participants, aged from 72 to 87 years performed and fulfilled the criteria for a spirometry, see below. The participants underwent a physical and medical examination including medical history and anthropometrics by a physician, and information was retrieved from medical records and a self-reported questionnaire including sociodemographic variables and lifestyle factors.

A three Tesla MRI examination (General Electric, discovery MR 750w) included four different images, a) an axial T2-weighted fluid-attenuated inversion recovery (T2 FLAIR); b) two settings of axial susceptibility-weighted angiography sequencies (SWAN) with 3 and 5 mm thick SWAN images to differentiate blood from calcifications; c) axial diffusion-weighted images (DWI); and d) sagittal T1-weighted 0.9-mm isotropic 3D fast spoiled gradient echo (3D-FSPGR) images, reconstructed in the axial and coronal planes. All MR images at baseline was assessed by one and the same experienced neuroradiologist (one of the authors, KAK) blinded for clinical information. WMH changes analyzed from the FLAIR sequences, were categorized according to the Fazekas scale, and a score of ≥ 2 was considered pathological for WMH.^13^ Lacunar infarctions were defined as having one or more ischemic infarction less than 1.0 cm in size located in the deep white matter, pons or the basal ganglia regions.^13^ Cerebral microbleeds (CMB) were defined as having one or more 0.2 to 0.5 cm hypointense lesion/s^14^ using the SWAN sequence. Medial temporal atrophy (MTA) was graded according to Scheltens’18 scale, and parietal and global cortical atrophy (GCA) were categorized according to the Koedam and Pasquier scales.^15,16^ Atrophy in specific regions like frontal cortical, temporal cortical and frontotemporal atrophy were assessed. A modified STRIVE variable from ‘Standards for reporting vascular changes on neuroimaging’ (STRIVE and STRIVE-2) was defined as presence of at least one of the following five criteria: Fazekas scale ≥ 2, ≥ 1 lacunar infarct, ≥ 1 CMB, cortical atrophy, and specific atrophy as presented by Wardlaw and co-authors.^17,18^

Spirometry was assessed by the Vitalograph model 2120 spirometer using the Spirotrac IV software (Vitalograph, Buckingham, UK). The spirometry was calibrated daily by trained experienced nurses and assessment was done according to ATS guidelines^19^ at all examinations. A bronchodilator 1.0 mg of β_2_-receptor agonist terbutaline was administrated 10 min prior to the spirometry and the subject was tested with a nose clip while seated. To interpret the quality of spirometry assessment, the Spirotrac software has a built in ATS test quality criteria with automatic feedback to the technician as a guidance. The goal was to conduct at least three acceptable spirometry curves and up to a maximum of eight consecutive breathing maneuvers were done. The forced expiratory volume in one-second (FEV_1_) from the best three attempts was recorded. Reproducibility criteria was used as an indication of more maneuvers were necessary as a standardization of spirometry.^19^

The reference equations for lower limit of normal (LLN) are available for subjects up to 95 years of age. ‘The 2012 Global Lungs Initiative (GLI) reference equations’ were used to calculate z-scores for FEV_1_ and FVC.^20^ Information on diagnosis of COPD came from the patients’ medical records and the county Region Skåne Healthcare Registry.^21^ Information on drug use was retrieved from medical records and the self-reported questionnaire. The three clinical criteria of COPD diagnosis from the Region Skåne Healthcare Registry are as follow a FEV1/FVC quotient less than 0.7 after bronchodilation, current airway symptoms, and a history of a risk factor for COPD.^21^

Estimated glomerular filtration (eGFR), adjusted for age and sex, was derived from the CKD-EPI formula, ‘the chronic kidney disease epidemiology collaboration’ based on creatinine and cystatin C assessments.^22^ CKD is defined as eGFR less than 60 ml/min/1.73 m^2^. Coronary artery disease covered the following conditions: myocardial infarction, heart failure, angina pectoris, atrial fibrillation and presence of cardiac vascular implants. Stroke/TIA included nontraumatic intracranial hemorrhage, cerebral infarction, and transient ischemic episodes (TIA). All diagnoses were coded according to the International Classification of Diseases, ICD-9, and ICD-10 and derived from the medical history, examination and medical records, and the National Inpatient and Outpatient Register to avoid recall bias.

Information on lifestyle factors was retrieved from the self-reported questionnaire. Smoking habits were categorized into three groups: regular/occasional smoking, former smokers, and nonsmokers. Levels of physical activity was dichotomized in two groups, a low to medium level comprising sedentary lifestyle or light activity for two to four hours weekly, and high-level including gardening, running or other exhausting activities for three or more hours weekly. Alcohol intake the past year was divided in: several times a week, less than once a week and abstainer. Cognitive function was assessed by the Mini-Mental State Examination (MMSE) were a score less than 24 denote impaired cognition, score between 24 to 27 an intermediate reduced cognition and scores between 28 to 30 a normal cognitive function. Only three subjects had a MMSE score below 24 indicating that the study population represented a general healthy population.

The statistical program IBM SPSS version 27 was used for all statistical analyses. Chi square tests were used to compare baseline characteristics. A logistic multiple regression model was used to calculate odds ratios using FEV_1_ z score ≥ 0 and respectively FVC z score ≥ 0 as the reference groups, adjusted for age. The z-scores for FEV_1_ and FVC and FEV_1_ / FVC quotient showed normal distribution and were tested for skewness. Comparison between statistical significance level was set at 5%. The STROBE checklist for cohort studies was used.

## Results

379 participants aged 72 to 87 years completed brain MRI and spirometry. Baseline characteristics of demography, lung function and comorbidities are summarized in Table 1 and categorized by FEV_1_ z scores. Distribution of gender, education and alcohol intake did not differ between groups categorized according to FEV_1_ z score. As expected, the group with the lowest FEV_1_ level had higher proportion of former smokers. With decreased lung function, expressed by lower z-score categories, the proportion of the covariate's hypertension, hyperlipidemia, smoking and low physical activity increased.

**Table 1.** Baseline characteristics at the examination in relation to z FEV_1_ of participants from the general population study Good Aging in Skåne (GÅS), part of the Swedish National study on Aging and Care. Chi square test was used to test group differences.

Study population (n = 379)	z FEV_1 _≥ 0	z FEV_1 _< 0 to −1	z FEV_1 _< −1.0	
	n = 142	n = 153	n = 84	p
Age (mean/range)	76.7 (73–86)	77.1 (72–87)	76.7 (72–86)	ns
Men / Women (%)	41/59	44/56	46/54	ns
Education (%)				ns
Elementary school	34	35	29	
Secondary school	30	37	46	
University > 1 y	36	28	25	
Smoking (%)				<0.001
Never smoker	6	11	19	
Former smoker	49	47	60	
Current smoker	45	42	21	
Alcohol intake (%)				ns
Teetotaller	10	11	6	
Less than once a week	13	9	12	
Several times a week	77	80	82	
Physical activity past year (%)				0.04
Mostly sedentary	6	9	15	
Light activity	84	83	80	
Strenuous activity	10	7	5	
BMI (%)				ns
< 20 underweight	1	2	1	
25–29 overweight	46	49	49	
≥ 30 obese	18	20	20	
Diabetes, type 1 or 2 (%)	18	13	23	ns
Hypertension (%)	35	50	58	<0.001
Hyperlipidemia (%)	37	47	57	0.01
Coronary artery disease (%)	30	39	43	ns
Stroke/TIA (%)	14	11	12	ns
COPD (%)	6	18	48	<0.001
Cognition, MMSE (%)				ns
28–30	68	68	66	
24–27	27	29	32	
<24	5	3	2	
SBP/DBP mm Hg mean ; SD	135 / 76; 15.2 / 8.5	136 / 76; 15.6/9.3	135 / 75; 16.5/9.2	ns/ns
eGFR^a^ mean /SD ml/min/1.73m^2^)	70.0 / 12.1	67.8 / 13.6	67.7 / 14.0	ns
% eGFR < 60 ml/min/1.73m^2^	15	28	28	0.015
FEV_1_ mean / SD	2.75 / 0.59	2.27 / 0.47	1.77 / 0.48	-
FVC mean / SD	3.38 / 0.85	2.94 / 0.67	2.60 / 0.75	ns
FEV_1_ / FVC	0.79 / 0.61	0.76 / 0.70	0.71 / 0.93	ns
% < LLN^a^ (z score < 1.64)	0	0	37	<0.001

^a^ LLN = lower limits of normality

Regression analyses of FEV_1_ and FVC z scores related to MRI markers of CSVD, adjusted for age, are presented in Table 2. Decreased lung function, both FEV_1_ and FVC z scores below ≤ −1.0, were associated with increased risk of WMH and GCA. The association with WMH was noted for women, but not for men. Decreased FVC z scores were also associated with MTA in women and precuneus atrophy in men. The modified STRIVE score, a MRI marker of vascular changes, was also associated to low FVC score in women alone.

**Table 2.** Odds ratios (OR) for MRI markers of cerebral small vessel disease in relation to z FEV_1_ and z FVC categories, stratified for gender and adjusted for age.

Total study sample	z FEV_1 _≥ 0 143		z FEV_1 _< 0 to −1 153	z FEV_1 _< −1.0 84		zFVC ≥0 141	zFVC < 0 to −1 161		zFVC < −1.0 78	
Men (%) / Women (%)	58 (41) / 85 (59)		67 (44) / 86 (56)	39 (46) / 45 (54)		48 (34) / 93 (66)	70 (43) / 91 (57)		46 (59) / 32 (41)	
MRI findings	HR p	HR	p	HR	p	HR p	HR	p	HR	p
		56 (43) / 73 (57)	7.02/7.01		393/512	-				
WMHs^a^ (all)		1.11	0.700	1.98	**0** **.** **021**		0.817	0.448	2.41	**0**.**003**
Men	reference	1.06	0.892	1.01	0.986	reference	0.89	0.790	1.53	0.360
Women		1.15	0.684	3.44	**0**.**002**		0.81	0.530	5.88	**<0**.**001**
Lacunar infarction^b^		1.22	0.649	1.39	0.510			**0**.**014**	1.47	0.535
Men	reference	0.82	0.748	0.47	0.376	reference	2.24	0.247	0.66	0.652
Women		1.80	0.362	3.09	0.095		4.21	**0**.**032**	3.00	0.194
CMBs^c^		1.25	0.408	1.09	0.783		1.32	0.299	1.32	0.383
Men	reference	1.45	0.336	0.88	0.781	reference	1.37	0.445	1.36	0.486
Women		1.05	0.904	1.29	0.566		1.19	0.623	0.96	0.938
Global cortical atrophy^d^		1.77	0.147	2.80	**0**.**014**		1.46	0.329	2.67	**0**.**018**
Men	reference	1.02	0.966	2.56	0.075	reference	0.98	0.972	2.00	0.196
Women		3.66	0.056	3.42	0.103		1.95	0.248	2.53	0.189
MTA^e^		1.00	0.990	1.53	0.198		1.23	0.431	2.11	**0**.**037**
Men	reference	0.79	0.599	0.76	0.599	reference	0.63	0.341	0.66	0.440
Women.		1.08	0.816	2.40	0.054		1.48	0.226	570	**0**.**007**
Specific atrophy^f^		0.87	0.577	0.63	0.133		0.90	0.675	0.60	0.114
Men	reference	0.94	0.871	0.57	0.255	reference	1.40	0.412	0.65	0.381
Women.		0.83	0.575	0.68	0.343		0.70	0.256	0.69	0.417
Parietal atrophy		1.18	0.508	1.36	0.305		1.24	0.381	0.98	0.940
Men	reference	0.97	0.934	1.16	0.735	reference	0.91	0.815	0.74	0.495
Women.		1.39	0.342	1.48	0.337		1.58	0.177	1.03	0.951
Precuneus atrophy		1.28	0.360	1.97	**0**.**024**		0.97	0.918	1.63	0.107
Men	reference	0.99	0.983	2.22	0.071	reference	0.91	0.826	2.54	**0**.**037**
Women		1.58	0.196	1.68	0.215		1.07	0.830	0.90	0.815
WMC pontine		1.25	0.491	1.42	0.344		1.00	0.987		0.249
Men	reference	1.56	0.389	1.08	0.898	reference	1.30	0.663	2.32	0.157
Women		1.07	0.867	1.74	0.230		0.93	0.850	1.29	0.624
Modified STRIVE^g^		1.61	0.087	1.16	0.632		1.10	0.714	1.46	0.280
Men	reference	2.32	0.065	0.68	0.388	reference	1.71	0.222	1.08	0.868
Women		1.27	0.504	2.22	0.100		0.85	0.622	4.87	**0**.**041**

^a^ WMHs white matter hyperintensities defined as Fazekas scale ≥ 2.

^b^ Lacunar infarcts defined as presence of ≥ 1 ischemic infarction (< 1.0 cm).

^c^ CMBs cerebral microbleeds defined as defined as presence of ≥ 1 small hypointense lesion (0.2–0.5 cm).

^d^ Global cortical atrophy (GCA) ≥ 1 according to Pasquier scale.

^e^ MTA medial temporal lobe atrophy.

^f^ Specific atrophy defined as at least one of the following entities global cortical atrophy (GCA) ≥ 1 according to Pasquier scale, Koedam parietal score ≥ 1. frontal/frontotemporal/temporal atrophy and ≥ 1.

^g^ Presence of CSVD according to the modified STRIVE variable was defined as the presence of at least one of the following entities Fazekas scale ≥ 2, ≥ 1. lacunar infarct, ≥ 1 CMB, cortical atrophy and specific atrophy.

Lacunar infarction was associated with decreased FVC z scores below 0 to −1 but not below ≤ −1.0. There were no gender differences for the baseline characteristics except for higher physical activity (44% versus 34%) and lower proportion of coronary artery disease among women (32% versus 43%) compared to men.

We have previously reported about the co-occurrence but high heterogeneity between CSVD manifestations and likewise in this study the modified STRIVE score was correlated to WMH, lacunar infarction, CMB, MTA, specific atrophy and WMC pontine with correlation coefficients in the range from 0.11 to 0.42.^23^

## Discussion

Lower lung function was associated with markers of CSVD, like WMH, lacunar infarctions and atrophies, particularly for women.

Previous studies have shown that reduced lung function like FEV_1_ and FVC are associated with cognitive decline.^24^ A cross-sectional study of participants in late life found an association with lower WMH volume, in line with our study.^6^ However, findings are inconsistent. In another MRI study examining cognitively normal individuals, the lowest quintile of FVC was associated with WMH and lacunes, but not with cerebral atrophies, and no associations were found between CVSD markers and FEV_1_.^25^ In a six community-based multi-center study, positive associations were noted between both FEV_1_ and FVC and lower brain volume, hippocampal volume and WMH, but not between the quotient FEV_1_/FVC and WMH.^6^ Reduced FEV_1_ is related to COPD, a known risk for CSVD, whereas reduced FVC as an expression of restrictive lung function, is associated to cardiovascular disease, diabetes, metabolic syndrome and inflammation, all of them potential risk factors for CSVD.^26^

Only a few studies have so far investigated the relationship between lung function and brain atrophies.^6,7,25^ As mentioned earlier, the Cross-Cohort Collaboration Consortium including six community base studies^6^ reported overall associations for FEV_1_ and FVC with lower hippocampal volumes and WMH, in line with our results, showing lower GCA, MTA, precuneus atrophy and WMH with reduced lung function. In contrast to our study, previous studies have not presented gender specific data. A recent study from the UK Biobank imaging study including more than 45,000 participants on clinical phenotypes related to CSVD and total WMH volume as the independent variable, identified respiratory problems, adjusted for age and sex, supporting our MRI findings.^27^

Female sex is a risk factor for having a restrictive spirometry pattern with reduced FVC but preserved FEV_1_/FVC.^28^ We have previously reported that female sex was associated to a higher relative lung function decline, adjusted for sociodemographic factors and smoking habits in the general elder population.^29^ A gender difference with a higher WMH burden in older females and an acceleration in WMH volume increase in females over the age 65 to 70 years was reported by the 15 general population-based cohort study.^9^ Postmenopausal status has been one explanation for the gender difference in CSVD manifestations and another explanation could be a higher pulmonary susceptibility to cardiovascular risk factors in women.^28^ Similar sex-specific findings were noted for WMH in a study of participants with mild cognitive impairment and dementia.^8^ The reduced FEV_1_ and FVC with WMH, lacunar infarction and MTA in this study might indicate a risk association between CSVD and reduced lung function possibly explained by related shared risk factors.

Strengths of this study include representation of both genders, a study sample from the general elder population with an age range from 72 to 87 years, and standardized and established protocols for spirometry and MRI assessments. There are limitations to this study. Established scales were used to grade WMH and atrophies by one and the same experienced neuroradiologist, but intra-rater variability was not assessed why the accuracy of the MRI markers and individual difference in assessment cannot be ruled out. No WMH volumes were calculated and information on tract specific WMH is therefore lacking. The lack of association between lacunar infarction and z FVC in the lowest category was most likely due to less statistical power in that group. The cross-sectional design will be unable to establish causality. Finally, the study population reflects a Caucasian population which limits generalizability to other ethnicities.

In conclusion, lower lung function expressed by reduced FEV_1_ and FVC z scores were associated with MRI markers of CSVD in this general healthy population and the association was particularly strong with WMH within CSVD, especially in women. Although possible shared risk factors exist between lung and heart disease, lung function should be recognized in future studies on CSVD.
